# Circulating Soluble ACE2 Plays an Independent Role to Protect against Vascular Damage in Diabetic Mice

**DOI:** 10.3390/antiox11050987

**Published:** 2022-05-18

**Authors:** Chris Tikellis, Gardner N. Robinson, Carlos J. Rosado, Duygu Batu, Maria A. Zuniga-Gutierrez, Raelene J. Pickering, Merlin C. Thomas

**Affiliations:** Department of Diabetes, Central Clinical School, Monash University, Melbourne, VIC 3004, Australia; christos.tikellis@monash.edu (C.T.); gardner.robinson@monash.edu (G.N.R.); carlos.rosado@monash.edu (C.J.R.); duygu.batu@nh.org.au (D.B.); alejandra.zunigagutierrez@monash.edu (M.A.Z.-G.); raelene.pickering@monash.edu (R.J.P.)

**Keywords:** Angiotensin-converting enzyme 2 (ACE2), angiotensin II, atherosclerosis, diabetes, DNA minicircle

## Abstract

Circulating levels of soluble ACE2 are increased by diabetes. Although this increase is associated with the presence and severity of cardiovascular disease, the specific role of soluble ACE2 in atherogenesis is unclear. Previous studies suggested that, like circulating ACE, soluble ACE2 plays a limited role in vascular homeostasis. To challenge this hypothesis, we aimed to selectively increase circulating ACE2 and measure its effects on angiotensin II dependent atherogenesis. Firstly, in *Ace2/ApoE* DKO mice, restoration of circulating ACE2 with recombinant murine soluble (rmACE2_19-613_; 1 mg/kg/alternate day IP) reduced plaque accumulation in the aortic arch, suggesting that the phenotype may be driven as much by loss of soluble ACE2 as the reduction in local ACE2. Secondly, in diabetic *ApoE* KO mice, where activation of the renin angiotensin system drives accelerated atherosclerosis, rmACE2_19-613_ also reduced plaque accumulation in the aorta after 6 weeks. Thirdly, to ensure consistent long-term delivery of soluble ACE2, an intramuscular injection was used to deliver a DNA minicircle encoding ACE2_19-613_. This strategy efficiently increased circulating soluble ACE2 and reduced atherogenesis and albuminuria in diabetic *ApoE* KO mice followed for 10 weeks. We propose that soluble ACE2 has independent vasculoprotective effects. Future strategies that increase soluble ACE2 may reduce accelerated atherosclerosis in diabetes and other states in which the renin angiotensin system is upregulated.

## 1. Introduction

Activation of the renin angiotensin aldosterone system (RAAS) is implicated in the development and progression of atherosclerotic cardiovascular disease (ASCVD) in diabetes, leading to an increased risk for heart attack, stroke, amputation, and premature mortality. Angiotensin (Ang) II is a vasoconstrictor and triggers renal sodium/fluid retention. Beyond its effects on blood pressure/electrolyte regulation, Ang II also directly modulates oxidative stress, inflammation, and endothelial dysfunction that accelerates atherogenesis in the vasculature [1,2,3,4,5]. Angiotensin-converting enzyme 2 (ACE2) has a counterregulatory role degrading angiotensin (Ang) II to generate Ang 1–7, a peptide with vasodilatory and anti-atherogenic activities that counterbalance those of Ang II, generated by Angiotensin-converting enzyme (ACE) [6,7]. In diabetes, the induction of sheddases increases ACE2 shedding from the endothelial surface [8,9]. This results in elevated circulating levels of ACE2 [10] that are correlated with the presence and severity of diabetic complications, including ASCVD [10,11]. The association between circulating soluble ACE2 and the risk for ASCVD may be an indirect biomarker of the depletion of ACE2’s atheroprotective activity in the diabetic vasculature following local shedding. Plasma ACE2 has recently been shown to be associated with increased risk of major cardiovascular events [12,13]. Alternatively, elevated circulating ACE2 may be an important counter-regulatory defence mechanism to mitigate the systemic effects of RAAS activation in diabetes. Consistent with this latter hypothesis, in this paper we show that selectively increasing circulating soluble ACE2 using two different approaches, either recombinant murine ACE2 (rmACE2) or intramuscular injection of DNA minicircles encoding soluble ACE2, attenuates vascular inflammation, oxidative stress, and atherosclerosis in a susceptible pro-atherosclerotic murine model.

## 2. Materials and Methods

*Model 1:* Treatment with recombinant murine ACE2: Male *ACE2/ApoE* double knockout mice (DKO) and *ApoE* KO controls aged 8-weeks, weighing between 20–25 g, were randomly allocated to receive recombinant murine ACE2 (rmACE2; APEIRON Biologics AG, Vienna) at a dose of 1 mg/kg/every second day by intraperitoneal injection for 6 weeks. All groups were then followed for 6-weeks (until ~14 weeks of age).

*Model 2:* Diabetes: Male *ApoE* KO mice aged 8-weeks, weighing between 20–25 g, were randomly allocated for the induction of diabetes using streptozotocin (55 mg/kg, intraperitoneal for 5 days) with control mice receiving citrate buffer alone. After one week, control and diabetic *ApoE* KO mice were further randomised to receive recombinant murine ACE2 (rmACE2; APEIRON Biologics AG, Vienna) at a dose of 1 mg/kg/every second day by intraperitoneal injection for 6 weeks. All groups were then followed for 6-weeks (until ~14 weeks of age).

*Model 3:* DNA minicircle: A third study, control and diabetic *ApoE* KO mice were further randomised to receive a DNA minicircle encoding murine ACE2_19-613_ at a dose of 40 μg/hind leg by intramuscular injection. Minicircles were generated using a parental plasmid (System Biosciences, Palo Alto, CA, USA, catalogue number: MN502A-1) encoding murine ACE2_19-613._ The plasmids were extracted and purified using the QIAprep^®^ Spin Miniprep Kit (250) (QIAGEN, Hilden, Germany, catalogue number: 27106) as per the manufacturer’s instructions. After sequence confirmation, the plasmid was transferred into ZYCY10P3S2T *E. coli* (System Biosciences, Palo Alto, CA, USA, catalogue number: MN900A-1) using a heat shock method, and minicircle DNA was extracted and purified using the EndoFree^®^ Plasmid Mega Kit (QIAGEN, Hilden, Germany, catalogue number: 12381) as per the manufacturer’s instructions. All groups were then followed for 10-weeks (until ~18 weeks of age). *n* = 6–10 mice per group for all models.

*Care of animals, ethics, and approval:* Throughout the study, animals were given access to standard mouse chow (Animal Resources Centre, Perth, Australia) and water *ad libitum*. All experiments were approved by the animal ethics committee of the Alfred Medical Research Precinct and conform to the Guide for the Care and Use of Laboratory Animals published by the US National Institutes of Health (NIH Publication No. 85-23, revised 1996).

*Blood pressure:* Systolic blood pressure was measured using an IITC Life Sciences non-invasive Tail Cuff Blood Pressure Machine. Briefly, mice were placed in prewarmed holders and allowed to climatise before three blood pressure readings were taken with the average of the three readings used.

*Fasting blood glucose and glycated haemoglobin:* Fasting blood glucose was measured in a drop of blood using an automated system (Abbott Architect ci8200, Abbott Laboratories, Abbott Park, IL, USA). Red blood cells were separated from plasma by centrifugation of heparinized whole blood for measurement of glycated haemoglobin (GHb) using the cobas b 101 by ROCHE diagnostics.

*Plaque Area Quantitation*: The primary outcome of the study was plaque area in the arch of the aorta after Sudan IV staining. Plaque area was quantitated as described previously [4].

*ACE2 quantification:* To determine the quantity of ACE2 produced in cell media and in plasma, a mouse ACE2 ELISA Kit (CusaBio, Wuhan, Hubei, China, catalogue number: CSB-E17204m) was performed on samples as per the manufacturers’ protocol. To determine the enzymatic activity of the ACE2 produced in cell media and in plasma, we used a mouse ACE2 Activity Assay Kit (Fluorometric), Product number K897 BioVision, Milpitas, CA, USA.

*Measurement of gene expression:* To measure the expression of key atherogenic markers, three micrograms of total RNA were extracted from each aorta and used to synthesize cDNA with Superscript First Strand synthesis system for RT-PCR (Gibco BRL, Grand Island, NY, USA). Inflammatory and oxidative stress gene expression levels were measured and analysed by real-time quantitative RT-PCR performed with the TaqMan system (Applied Biosystems). Each sample was tested in triplicate and results were expressed relative to *ApoE* KO aortae, which were arbitrarily assigned a value of 1. PCR Primer sets used in the study (5′-3′): Mouse CD11b (NM_008401): Probe- 6-FAM ACTCTGCGTTTGCCCTG, Forward- GAGCAGCACTGAGATCCTGTTTAA, Reverse- ATACGACTCCTGCCCTGGAA; Mouse Interlukin-6 (IL-6; NM_031168): Probe- 6-FAM-ATTGCCATTGCACAACT, Forward- GGGAAATCGTGGAAATGAGAAA, Reverse- AAGTGCATCATCGTTGTTCATACA; Mouse Heme oxygenase-1 (Ho-1; NM_010442): Probe- 6-FAM-CTAAGACCGCCTTCCT, Forward- AGATGACACCTGAGGTCAAGCA, Reverse- TTGTGTTCCTCTGTCAGCATCAC; Mouse Krupple-like factor 3 (KLF3; NM_008453): Forward- TGACCACCTTGCCCTACACA, Reverse- TGAGCTGGAGACAGGTTTTCAG; Mouse Monocyte chemoattractant protein-1 (MCP1; NM_011333): Probe- 6-FAM-AATGGGTCCAGACATAC, Forward- GTCTGTGCTGACCCCAAGAAG, Reverse- TGGTTCCGATCCAGGTTTTTA; Mouse Tumour necrosis factor alpha (TNF-α; NM_013693): Probe- 6-FAM-TCACCCACACCGTCAG, Forward- GGCTGCCCCGACTACGT, Reverse- TTTCTCCTGGTATGAGATAGCAAATC; Mouse Vascular cell adhesion molecule-1 (VCAM-1; NM_011693): Probe- 6-FAM-CCAAAATCCTGTGGAGCAG, Forward- CTGCTCAAGTGATGGGATACCA, Reverse- ATCGTCCCTTTTTGTAGACATGAAG.

*Urinary albumin:* Urinary albumin excretion was estimated from the daily urine volume obtained during metabolic caging multiplied by urine volume concentration measured using a mouse albumin ELISA quantitation kit (Bethyl Laboratories, Montgomery, TX, USA).

*Statistics:* Continuous data are expressed as mean ± SEM. Differences in the means among groups were compared using 2-way ANOVA with *ApoE*/*ACE2* DKO and *ApoE* KO groups as the 2 variables. Pair-wise multiple comparisons were made with the Student–Newman–Keuls posthoc analysis to detect significant differences between groups. *p* < 0.05 was considered statistically significant.

## 3. Results

### 3.1. Recombinant ACE2 Attenuates Atherosclerosis in ACE2/ApoE Double Knockout Mice

**Figure 1:**
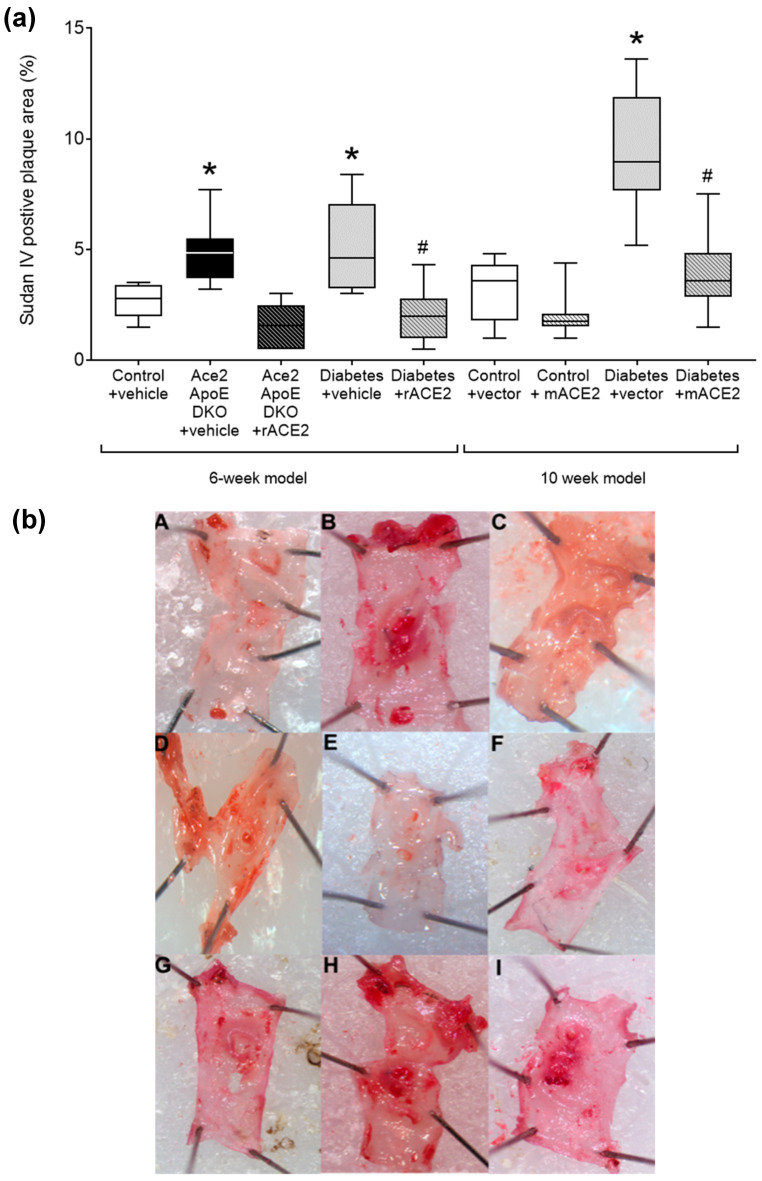
Quantified area of Sudan IV positive plaque area in the arch of the aorta (**a**). Data shows median ± 5–95% CI. Box shows the interquartile interval (25–75% percentiles); * *p* < 0.05 vs. vehicle/vector treated pair; ^#^ *p* < 0.05 vs. diabetes. Micrograph of the aortic arch for each group (**b**): (**A**)—Control + Vehicle; (**B**)—*Ace2/ApoE* DKO + Vehicle; (**C**)—*ACE2/ApoE* DKO + rmACE2; (**D**)—Diabetes + Vehicle; (**E**)—Diabetes + rmACE2; (**F**)—Control + Vector; (**G**)—Control + ACE2 minicircle; (**H**)—Diabetes + Vector; (**I**)—Diabetes + ACE2 minicircle, *n* = 6–10 mice per group.

**Figure 2:**
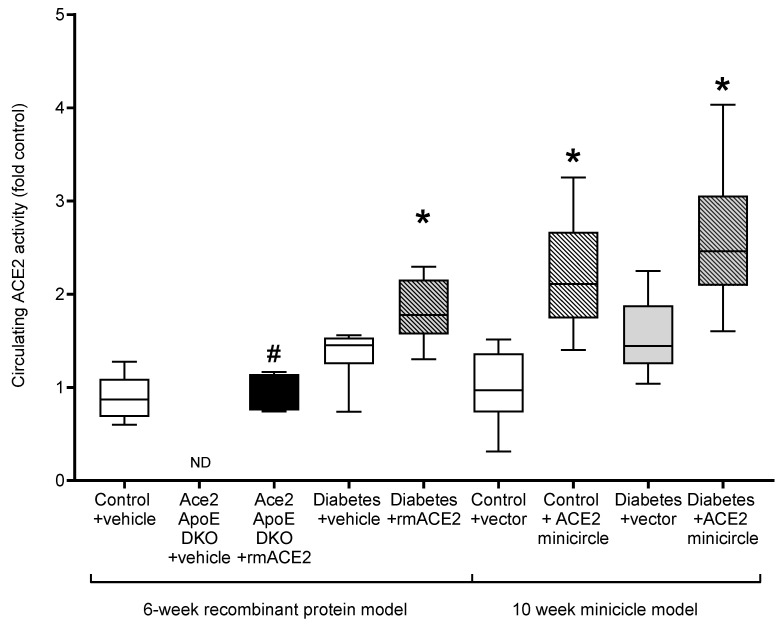
Circulating ACE2 activity in the plasma at endpoint, except for *Ace2/ApoE* DKO mice treated with rmACE2 where data shows ACE2 activity after 1 week of treatment (denoted by #). Data shows median ± 5–95% CI. Box shows the interquartile interval (25–75% percentiles); * vs. vehicle/vector treated pair, *p* < 0.05. *n* = 6–10 mice per group. ND: not detected.

As previously reported [14], *Ace2/ApoE* DKO mice have increased atherosclerotic plaque accumulation (Figure 1). To specifically examine the role of the loss of soluble ACE2 in this model, *Ace2/ApoE* DKO mice were treated with recombinant murine ACE2 (rmACE2; 1 mg/kg/alternate day IP). Having never been exposed to ACE2, this protein is immunogenic in this setting. We were able to transiently increase circulating ACE2 into the physiological range using this protocol (Figure 2), although this increase was not sustained, possibly due the development of circulating antibodies in *Ace2* KO mice which confound immune-based analysis. Nonetheless, this modest increase in circulating ACE2 following treatment with rmACE2 was associated with a reduction in the accumulation of Sudan IV positive plaque in the arch of the aorta in *Ace2/ApoE* DKO mice when compared to untreated mice (Figure 1). Other parameters, including weight and systolic blood pressure were not affected by rmACE2 in *Ace2/ApoE* DKO mice (Table 1).

### 3.2. Recombinant ACE2 Attenuates Atherosclerosis in Diabetic ApoE Knockout Mice

To validate that increasing soluble ACE2 alone could also modulate diabetes-associated atherosclerosis, we treated diabetic *ApoE* KO mice with rmACE2 (1 mg/kg/alternate day IP) for 6 weeks. Diabetes is associated with activation of the RAAS and accelerated atherosclerosis that is prevented by RAAS blockade in this setting [4,5]. When treated with rmACE2 for 6 weeks, circulating ACE2 activity increased approximately two-fold in diabetic mice (Figure 2). This was also associated with a reduction in the accumulation of Sudan IV positive plaque in the arch of the aorta when compared to untreated diabetic mice (Figure 1), to levels not significantly different to non-diabetic *ApoE* KO mice. Again, systolic blood pressure, blood glucose, HbA1c, and body weight were not affected by rmACE2 (Table 1).

### 3.3. DNA Minicircles Encoding Soluble ACE2 Attenuates Atherosclerosis in Diabetic ApoE Knockout Mice

*ApoE* KO mice were injected (intramuscularly into two calf muscle beds) with DNA minicircles (40 μg/leg) encoding murine soluble ACE2_19-613_ (i.e., the catalytic domain of ACE2) to achieve long-term systemic delivery. Following the induction of streptozotocin diabetes, mice were followed for 10 weeks. This treatment was associated with an approximate two-fold increase in ACE2 activity, similar to that achieved by injection of rmACE2. This increase was sustained across the study period (Figure 2). After 10 weeks of follow up, the accumulation of Sudan IV positive plaque in the arch of the aorta was also reduced in diabetic mice receiving the ACE2 minicircle (Figure 1), to levels not significantly different from control *ApoE* KO mice.

Urinary albumin excretion was reduced in diabetic *ApoE* KO mice following treatment with an ACE2 minicircle (Mean ± SEM; Diabetes 48 ± 5 µg/day, Diabetes + minicircle 28 ± 3 µg/day, *p* = 0.008). However, systolic blood pressure, blood glucose, HbA1c, and body weight were not affected by rmACE2 (Table 1).

### 3.4. Recombinant ACE2 and DNA Minicircles Encoding Soluble ACE2 Attenuate Oxidative Stress and Inflammation in the Vascular Wall in Diabetic ApoE Knockout Mice 

**Figure 3:**
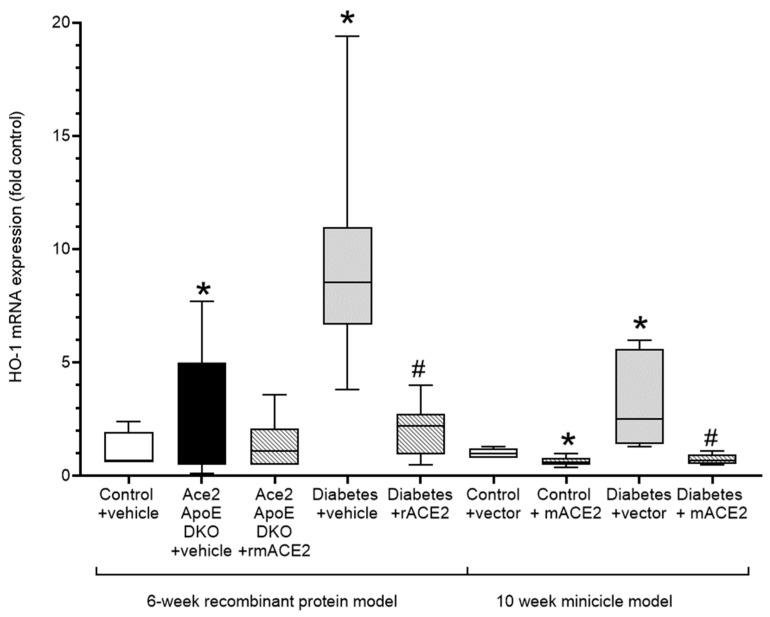
Expression of the Nrf-2 dependent oxidative stress marker, Heme-Oxygenase (HO-1) in the thoracic aorta. Data shows median ± 5–95% CI. Box shows the interquartile interval (25–75% percentiles); * *p* < 0.05 vs. vehicle/vector treated pair ^#^ *p* < 0.05 vs. diabetes. *N* = 6–10 mice per group.

Atherogenesis is characterised by the induction of oxidative stress and inflammation in the vessel wall that plays a major role in lipid retention and vascular dysfunction, leading to plaque accumulation. The upregulation of Nrf2-regulated genes including heme oxygenase-1 (HO-1) is both a biomarker of vascular oxidative stress and a compensatory vasculoprotective response. In our models, increases in diabetes-associated atherosclerosis was accompanied by a marked increase in vascular HO-1 expression. Treatment with rmACE2 or ACE2 minicircles significantly reduced expression of HO-1 consistent with their atheroprotective actions in this model (Figure 3).

Diabetes is also associated with upregulation of vascular inflammation including the induction of pro-atherogenic mediators in the thoracic aorta, including the adhesion molecule (VCAM-1) and inflammatory markers (TNFα, IL-6, MCP-1, CD11b, and KLF3). Consistent with their anti-atherosclerotic effects, the expression of each of these markers were decreased in diabetic mice following treatment with either rmACE2 or ACE2 minicircles (Table 2). 

## 4. Discussion

Activation of RAAS is an important driver in diabetic complications, including accelerated atherosclerosis leading to ASCVD and premature mortality [4,15]. Although circulating components of the RAAS are often considered secondary to the tissue or local RAAS, in this study we demonstrate that circulating soluble ACE2 likely plays an independent anti-atherosclerotic role. In particular, we show that increasing circulating ACE2 activity alone, even in the absence of tissue ACE2 suppresses atherogenic mediators in the vasculature, including oxidative stress [16] and inflammation [17], and reduces the development of atherosclerotic plaque.

We have previously shown that genetic deficiency of *Ace2* results in increased tissue and circulating levels of Ang II [14]. In susceptible models, this leads to modest hypertension [18], cardiac hypertrophy [19], accelerated aortic aneurysms [20], and augmented atherosclerosis in *ApoE* KO mice [14]. It has been hypothesised that cardiovascular damage in these conditions is predominantly due to the loss of ACE2 from cell surface membranes, exposing cells to increased local levels of Ang II. However, genetic *Ace2* deficiency also results in the loss of circulating soluble ACE2. We propose that the loss of soluble ACE2 may also be an important and independent determinant of the *Ace2* knockout phenotype, as in this study, we show that restoration of soluble ACE2 to physiological levels in *Ace2/ApoE* DKO mice can prevent the characteristic acceleration of atherosclerosis usually seen in this model and can be prevented even without restoration of tissue ACE2.

ACE2 is normally liberated from the endothelium into the circulation by sheddases, including ADAM-17, that are induced in diabetes by high levels of blood glucose, oxidative stress, and vascular inflammation [8,9]. We hypothesised that this may partly be a ‘defence mechanism’ to antagonise disease-associated activation of the RAAS. The preponderance of studies employing treatment with recombinant ACE2 have shown positive effects on cardiac hypertrophy [21] and hypertension [22]. In this study, we demonstrate that increasing circulating soluble ACE2 is also able to attenuate diabetes-associated atherosclerosis and markers of inflammation and leucocyte recruitment, as well as significantly reduce oxidative damage. This may be partly mediated by altering circulating systemic mediators of the RAAS, metabolising circulating Ang II, and generating Ang 1–7 [23]. However, experimental models of pulmonary hypertension also confirm a direct effect of soluble ACE2 on vascular remodelling [24] that may be relevant to the anti-atherosclerotic potential of soluble ACE2 as demonstrated in this study. 

Although we show that increased soluble ACE2 achieved by DNA minicircles can attenuate diabetes-associated atherosclerosis, this finding differs from diabetes-associated kidney damage, previously reported in diabetic mice on an FVB/N background, which showed that ACE2 minicircles did not prevent kidney injury or dysfunction associated with diabetes, despite a significant increase in circulating ACE2 [25]. This led the authors to conclude that serum ACE2 alone may not afford end-organ protection in diabetes. However, we believe that athero-protection afforded by circulating soluble ACE2 may differ from the kidney, as circulating soluble ACE2 is in direct contact with the intravascular environment and is thus likely to have greater potential to modify oxidative stress and inflammation associated with RAAS activation, and its subsequent contribution to leukocyte recruitment and adhesion to the nascent atherosclerotic lesion. In addition, we note that in our diabetic *ApoE* KO mice (on a C57Bl6 background) the induction of albuminuria was modestly reduced, albeit from a low level compared to the far more susceptible FVB/mice previously reported [25]. In our early and less severe kidney model, amelioration of endothelial dysfunction by soluble ACE2 may be sufficient to reduce albuminuria, while more severe renal lesions cannot be accessed by soluble ACE2 that is both too large to be filtered at the glomerulus and fails to modulate the highly-active intra-renal RAAS [25]. We also assayed 24-h urinary albumin excretion, as spot urine ACRs may be problematic in diabetic FVB mice due to altered urinary creatinine excretion [26]. 

In this study, to further elucidate the postulated atherosclerotic role of soluble ACE2, we employed an alternative approach to replete soluble ACE2. Specifically, we used DNA minicircles injected into muscle to deliver soluble ACE2 for long-term over-expression. By administering the minicircle via an isolated intramuscular injection, we have essentially employed the muscle as a continuous delivery engine for the secretion of soluble ACE2, with a single timepoint injection lasting for at least 10 weeks. This novel method is a highly practical means to increase circulating soluble protein without directly modulating tissue activity that may disrupt local homeostasis. In a previous study, Wysocki, et al. were also able to achieve and sustain high levels of ACE2 activity for a long period using intraperitoneal DNA minicircles [25]. However, the intraperitoneal route they used may also have induced changes in ACE2 expression in the aorta and liver that may have confounded the interpretation of their results. Although distant, the skeletal muscle proved an excellent pump for liberating soluble ACE2 into the circulation following a single injection with a DNA minicircle and achieved very similar results to recombinant ACE2 delivered alternate daily. 

While our study demonstrates proof-of-concept surrounding the vasculoprotective effects of ACE2, there were many limitations to our approach, including the immunogenicity of murine ACE2 in *ACE2* KO mice leading to the development of antibodies that prevented measurement of the ACE2 delivered and potentially attenuated its effect. Although *ApoE* KO mice are widely used to model atherogenesis, the early lesion more resembles fatty streak of the nascent atherosclerotic plaque than the complex lesion associated with human cardiovascular disease. In addition, we do not demonstrate changes in vascular protein or protein activity that are characteristic of more advanced models of longer duration. Nonetheless, the attenuation of nascent atherogenic changes in the aorta observed in these models following elevation of circulating ACE2, is consistent with independent vasculoprotective activity. 

## 5. Conclusions

In summary, ACE2 deficiency is associated with enhanced atherogenesis. We propose that rather than being a bystander, these changes partly reflect the loss of soluble ACE2, which has an anti-atherosclerotic action. In diabetic mice, a further increase in soluble ACE2 administered, either directly as a recombinant protein or via DNA minicircle, protects against accelerated atherosclerosis in the vascular wall and reduces albuminuria. These findings emphasize the potential utility of increasing circulating soluble ACE2 as a strategy to reduce vascular damage and dysfunction in the diabetic context.

## Figures and Tables

**Table 1:** Physiological parameters.

	Body Weight (g)	Blood Glucose (mmol/L)	HbA1c (%)	Systolic Blood Pressure (mmHg)
**6-week model**				
***ApoE* KO**	28 ± 0.5	10.9 ± 0.5	4.4 ± 0.1	104 ± 2
***ApoE/Ace2* DKO**	26.2 ± 0.4	11.2 ± 0.4	4.5 ± 0.1	107 ± 1 *
***ApoE/Ace2* DKO + rmACE2**	26 ± 0.5	11 ± 0.3	4.7 ± 0.3	106 ± 2
***ApoE* KO + Diabetes**	21 ± 1.0 *	25 ± 2.5 *	9.2 ± 0.2 *	108 ± 2 *
***ApoE* KO + Diabetes + rmACE2**	22 ± 2.0 *	28 ± 0.5 *	8.7 ± 0.9 *	107 ± 1 *
**10-week model**				
***ApoE* KO**	29 ± 0.9	12 ± 0.9	4.8 ± 0.2	106 ± 1
***ApoE* KO + ACE2 minicircle**	30 ± 1.0	10 ± 1.0	4.1 ± 0.1	106 ± 1
***ApoE* KO + Diabetes**	24 ± 0.8 *	30 ± 1.5 *	12.3 ± 0.6 *	108 ± 1
***ApoE* KO + Diabetes + ACE2 minicircle**	27 ± 0.8 ^#^	25 ± 3.0 *	10.0 ± 0.4 *	103 ± 4

Angiotensin-converting enzyme 2 (ACE2), apolipoprotein E knockout (*ApoE* KO); recombinant murine ACE2 (rmACE2). Data shows mean ± SEM, significance is *p* < 0.05, * vs. *ApoE KO*, ^#^ vs. Diabetes + *ApoE* KO.

**Table 2:** Expression of markers of inflammation in the mouse aorta.

	IL-6	TNFα	VCAM-1	MCP1	KLF3	CD11b
***ApoE* KO**	1.0 ± 0.2	1.0 ± 0.2	1.0 ± 0.1	1.0 ± 0.2	1.0 ± 0.2	1.1 ± 0.3
***ApoE/Ace2* DKO**	5.5 ± 1.9 *	1.4 ± 0.6	0.5 ± 0.2	4.4 ± 1.5 *	1.3 ± 0.8	1.8 ± 0.5
***ApoE/Ace2* DKO + rmACE2**	0.5 ± 0.1	2.7 ± 0.9	0.7 ± 0.4	0.9 ± 0.2	1.5 ± 0.6	0.8 ± 0.2
***ApoE* KO + Diabetes**	6.4 ± 2.2 *	3.6 ± 0.8 *	4.0 ± 1.0 *	3.7 ± 0.9 *	2.9 ± 0.7	3.9 ± 0.9 *
***ApoE* KO + Diabetes + rmACE2**	0.5 ± 0.2 *^#^	1.4 ± 0.5 ^#^	0.4 ± 0.1 ^#^	1.2 ± 0.2 ^#^	1.7 ± 0.4	2.3 ± 0.4
		**10-week model**				
***ApoE* KO**	1.0 ± 0.2	1.0 ± 0.2	1.0 ± 0.2	1.0 ± 0.2	1.0 ± 0.1	1.1 ± 0.1
***ApoE* KO + ACE2 minicircle**	0.3 ± 0.1 *	0.2 ± 0.1	0.7 ± 0.1	0.1 ± 0.01 *	1.6 ± 0.2	1.1 ± 0.2
***ApoE* KO + Diabetes**	1.9 ± 0.4 *	3.5 ± 1.2 *	3.7 ± 1.1 *	3.1 ± 0.8 *	2.7 ± 0.7 *	2.7 ± 0.6 *
***ApoE* KO + Diabetes + ACE2 minicircle**	0.6 ± 0.1 ^#^	0.8 ± 0.3 ^#^	0.8 ± 0.1 ^#^	0.3 ± 0.06 ^#^	1.6 ± 0.2	1.4 ± 0.1

Angiotensin-converting enzyme 2 (ACE2), apolipoprotein E knockout (*ApoE* KO); recombinant murine ACE2 (rmACE2). Data shows mean ± SEM, significance is *p* < 0.05 * vs. *ApoE KO*, ^#^ vs. Diabetes + *ApoE* KO.

## Data Availability

Data is contained within the article.
